# Simultaneous Measurement of Contractile Force and Ca^2+^ Concentration Distribution in Human iPS Cell-Derived Cardiomyocytes

**DOI:** 10.3390/s25247478

**Published:** 2025-12-09

**Authors:** Ryota Ikegami, Takuya Tsukagoshi, Kenei Matsudaira, Hidetoshi Takahashi, Thanh-Vinh Nguyen, Kentaro Noda, Ken’ichi Koyanagi, Isao Shimoyama

**Affiliations:** 1 Graduate School of Integrated Engineering, Toyama Prefectural University, Imizu 939-0398, Toyama, Japan; u378004@st.pu-toyama.ac.jp; 2 Department of Intelligent Robotics, Faculty of Information Engineering, Toyama Prefectural University, Imizu 939-0398, Toyama, Japan; k_noda@pu-toyama.ac.jp (K.N.); koyanagi@pu-toyama.ac.jp (K.K.); 3 Department of Mechano-Informatics, Graduate School of Information Science and Technology, The University of Tokyo, Tokyo 113-8654, Japan; 4 Department of Mechanical Engineering, Faculty of Science and Technology, Keio University, Yokohama 223-8522, Kanagawa, Japan; htakahashi@mech.keio.ac.jp; 5 The University of Tokyo, Tokyo 113-8654, Japan; i-shimoyama@pu-toyama.ac.jp; 6 Toyama Prefectural University, Imizu 939-0398, Toyama, Japan

**Keywords:** piezoresistive cantilever, iPS cell-derived cardiomyocytes

## Abstract

Human-induced pluripotent stem cell-derived cardiomyocytes (hiPSC-CMs) hold significant promise for regenerative medicine but exhibit immaturity relative to native cardiomyocytes. To make hiPSC-CMs more similar to mature cardiomyocytes, extensive research is being conducted from biochemical, electrochemical, mechanical, and physical perspectives. Quantitatively assessing their maturation is essential to evaluate improvements in cardiac cell function and clarify the impact of previous research. In this study, we present a high-speed sensing system that enables simultaneous, real-time measurement of cardiomyocyte contractile force, and intra- and extra-cellular Ca^2+^ dynamics. To enhance measurement precision, a visualization technique is incorporated to identify individual cardiomyocytes. This simultaneous evaluation system for cardiomyocyte contractility and various ion concentrations has the potential to become an effective and powerful foundational technology for assessing cardiomyocyte maturation and the regenerative medicine applications of IPSC-CMs. The ability to convert cardiomyocyte contractile force into single-cell force implies a more universal evaluation of the mechanical properties.

## 1. Introduction

Human-induced pluripotent stem cell-derived cardiomyocytes (hiPSC-CMs) are increasingly utilized as powerful in vitro tools for regenerative medicine, cardiotoxicity studies, and investigating cardiac function. However, substantial differences remain between hiPSC-CMs and adult human cardiomyocytes, posing barriers to clinical applications; in particular, the former are immature relative to the latter, and transplantation can lead to life-threatening arrhythmias [1,2]. To address this immaturity, numerous approaches have been explored, including modulating developmental signaling pathways, electrical or mechanical stimulation, and culturing on substrates with surface microstructures [3,4,5,6].

Accurately evaluating these maturation strategies requires a quantitative assessment of cardiomyocyte properties. While visual methods such as microscopy and biochemical techniques including electrophoresis or ELISA have long been used to study cellular structure and function, the heart’s essential function as a pump necessitates mechanical measurements to assess cardiomyocyte maturation effectively.

Various methods have been proposed to mechanically quantify cardiomyocyte contraction, including atomic force microscopy, nonlinear optical microscopy, single-cell traction force microscopy, fluorescent microbeads, and micropost arrays [7,8,9,10,11,12,13]; however, most of these approaches require the analysis of microscopic images and provide limited real-time information on contractile force. To overcome these limitations, we previously developed a feedback control system incorporating piezoresistive force sensors to measure cardiomyocyte contraction and apply mechanical stimulation based on the measured force [14,15,16]. The heart is thought to sense mechanical load and adjust its contractile force accordingly, as exemplified by the Frank–Starling law [17,18]. Investigating this phenomenon at the cellular level requires simultaneously assessing biochemical energy supply and the mechanical dynamics of contraction.

In this study, we present a cell measurement system that combines a MEMS-based piezoresistive force sensor for real-time cardiomyocyte contraction measurement with fluorescence imaging of the Ca^2+^ concentration distribution. By using the MEMS sensor for force acquisition, the microscopy setup can be fully dedicated to the fluorescence observation, maximizing the temporal and spatial resolution. Myofibril contraction is known to result from transient increases in Ca^2+^ concentration, and understanding the molecular mechanisms underlying this process requires a dynamic correlation of contraction and Ca^2+^ signaling in cardiac tissue [19,20,21,22,23]. In particular, a point of significant interest is distinguishing whether changes in contractile force arise from Ca^2+^ concentration or myofilament sensitivity. Therefore, simultaneous, high-resolution measurement of Ca^2+^ dynamics and contractile force at the single-cell and multi-cellular level is essential for investigating cardiomyocyte physiological function.

## 2. Materials and Methods

### 2.1. Measurement System

**Figure 1:**
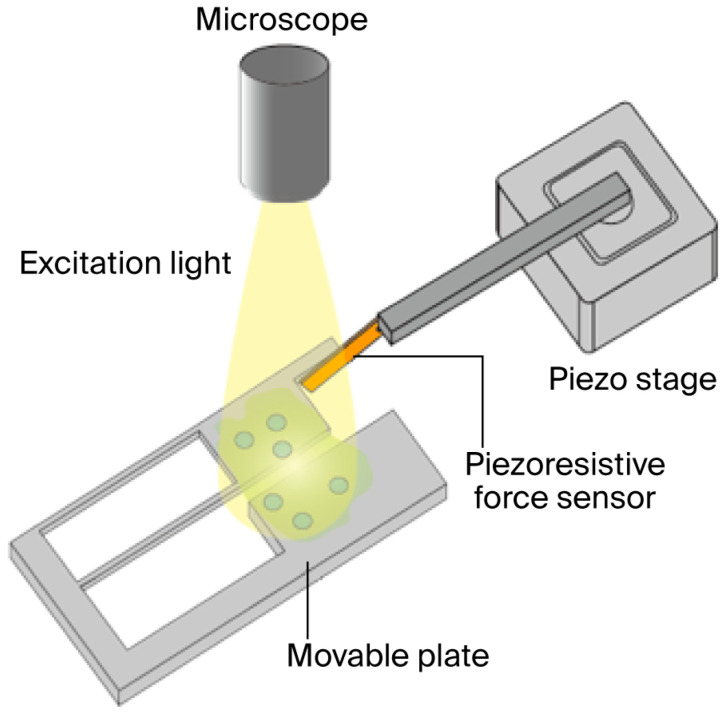
Equipment for simultaneously measuring contraction force and conducting fluorescent microscopy for hiPSC-CMs.

**Figure 2:**
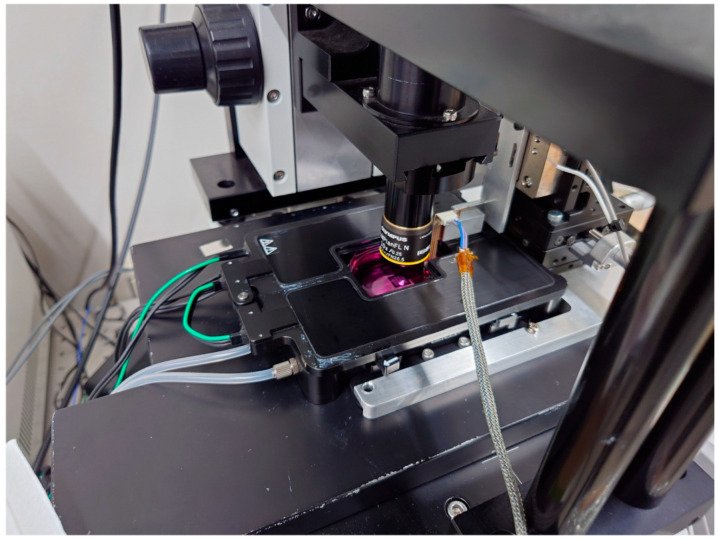
An incubator for hiPSC-CMs with movable plates, all of which are built up on the optical microscope.

In this study, the contractile force of hiPSC-CMs is measured using the system illustrated in Figure 1 and Figure 2. The hiPSC-CMs are pre-stained with a calcium fluorescent probe, enabling real-time observation of intra- and extra-cellular Ca^2+^ concentration distribution during force measurement using fluorescence microscopy. The measurement system is a custom-built platform designed specifically for evaluating hiPSC-CM contractile force, comprising a piezoresistive cantilever-type force sensor, a movable culture plate, a piezo stage for sensor positioning adjustment (B17-090, Nano Control Co., Ltd., Tokyo, Japan), a piezo controller (NCS6121C, Nano Control Co., Ltd., Tokyo, Japan), a Field-Programmable Gate Array (FPGA; cRIO-9030, National Instruments, Austin, TX, USA) that controls the piezo stage and acquires sensor data, and a host PC (Precision T3610, Dell Technologies Inc., Round Rock, TX, USA) used for managing the FPGA and recording the data [14,16].

The measurement system is built on an optical microscope, with a small incubator (custom-made, Tokai Hit Co., Ltd., Tokyo, Japan) installed on the microscope’s stage, which is equipped with a temperature controller (INU-UK-B18M, Tokai Hit Co., Ltd., Tokyo, Japan), a CO_2_ controller (GM-4000, Tokai Hit Co., Ltd., Tokyo, Japan), and a CO_2_ cylinder unit (MG1, Tokai Hit Co., Ltd., Tokyo, Japan), enabling the cells under measurement to be maintained in optimal condition.

HiPSC-CMs are seeded on a movable culture plate fixed to the bottom of a Petri dish and cultured for several days, during which the plate is fully immersed in culture medium, ensuring that the medium’s surface tension does not affect the cardiomyocytes. By inserting the objective lens from the top of the small incubator, the measured hiPSC-CMs can be observed under the microscope. The contraction of the hiPSC-CMs deforms the movable plate, which transmits force to the cantilever-type sensor. The sensor base is mounted on the piezo stage, enabling position adjustment as well as the application of mechanical stretch to the cardiomyocytes. The FPGA, controlled via LabVIEW (National Instruments, TX, USA), adjusts the piezo stage position and records signals from the force sensor.

### 2.2. Movable Plate

**Figure 3:**
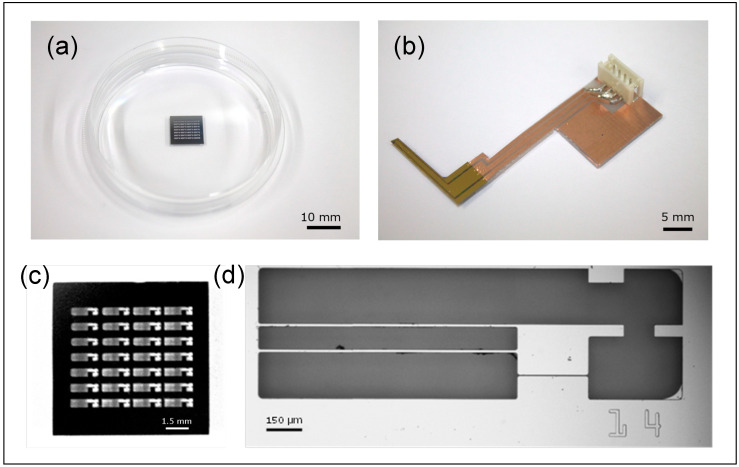
The silicon-based structures for measuring contraction forces of the hiPSC-CMs. (**a**) A total of 24 movable plates formed on a silicon wafer. (**b**) The piezoresistive force sensor fixed on a printed circuit board. (**c**) A Si wafer with 28 movable plates. (**d**) A single movable plate.

The movable culture plate, illustrated in Figure 3, is designed and fabricated to accommodate the size and contractile force of hiPSC-CMs [14,15,16]. It is made of single-crystal silicon, ensuring biocompatibility with cardiomyocytes, and consists of two components: a movable and a fixed part, separated by a 5 µm gap. The plate is fabricated using standard MEMS processes, with a total size of 1 cm × 1 cm, and contains 24 movable–fixed pairs. The fixed part is 253 µm thick and adheres to the dish bottom; the movable part is supported by two thin arms (length: 1100 µm; width: 10 µm; thickness: 2 µm) and can easily deform in response to hiPSC-CM contractions.

HiPSC-CMs seeded on the plate form a monolayer through inter-cellular connections, spanning the gap between the movable and fixed parts. Synchronously beating cardiomyocytes deform the plate’s movable section, and a protrusion on this section makes contact with the cantilever-type force sensor, enabling measurement of contractile force. While piezoresistive sensors can be fabricated with side doping to sense lateral forces, this complicates the fabrication process. In this study, the sensor is positioned laterally in contact with the protrusion, allowing reliable contractile force measurement while maintaining high yield and repeated measurements. In the future, side-doped plates may enable independent measurement and the application of mechanical loads to cells.

### 2.3. MEMS Force Sensor

The force sensor is a piezoresistive cantilever-type sensor [24,25,26,27,28] that exploits the piezoresistive effect, which involves a change in electrical resistance in response to mechanical strain. Appropriate doping of silicon allows for effect tuning. In thin cantilever structures, strain-induced elongation and compression are canceled on the top and bottom surfaces, so doping near the surface is crucial. Sensitivity is inversely proportional to the square of the cantilever thickness, and modern cantilevers can be as thin as 80 nm [29].

In this study, a circuit for converting the change in electrical resistance into a voltage signal is implemented in the sensing system to detect the voltage signal as data, and the relationship between voltage and force is pre-calibrated and stored as data [30]. During the measurements, the cantilever makes contact with the movable plate protrusion, and the displacement and contractile force are derived from the voltage signal. Because the sensor signal is extremely weak, an amplifier circuit is used to amplify it. The circuit is equipped with a high-pass filter to reduce baseline drift due to temperature fluctuations, convection in the culture medium, and experimental bench vibrations.

### 2.4. hiPSC-CM Seeding and Culture

MiraCell cardiomyocytes (from ChiPSC12, TAKARA BIO, Inc., Kusatsu, Shiga, Japan) were used, namely experimental human iPS cell-derived cardiomyocytes. Cells were thawed according to the manufacturer’s protocol and seeded onto the movable culture plate. Seeding and culture procedures are detailed below.

The day before seeding, the plate was sprayed with 70% ethanol and UV-sterilized for at least 24 h in a draft chamber. On the day of seeding, 70% ethanol was applied again, allowed to evaporate, and UV-sterilized. Fibronectin solution (50 µg/mL, 100 µL) was then applied to the plate and incubated at 37 °C with 5% CO_2_ for 2 h to promote adhesion, with excess fibronectin aspirated. Approximately 7.0 × 10^6^ cells/mL MiraCell CMs (70 µL) were seeded on the plate and incubated under the same conditions for 5 h to allow attachment. Subsequently, the plate was cultured in medium supplemented with 1% penicillin–streptomycin at 37 °C with 5% CO_2_.

Half of the medium was replaced every 2 days, and full replacement occurred the day before measurement. After approximately 4 days, the plate showed observable displacement due to contractions; measurements were performed on cells cultured for at least 5 days, with contractions maintained for up to 18 days.

### 2.5. Visualization of Ca^2+^ Concentration

The movable plate was fixed to a 5 mL Petri dish, and hiPSC-CMs were cultured as described in Section 2.4. Fluo4-AM (50 µg; Dojindo Laboratories, Kumamoto, Japan) was dissolved in 50 µL DMSO to prepare the Fluo4 solution, which was added to 10 mL of pre-warmed serum-free maintenance medium (STEMdiff Cardio Maintenance Basal Medium, Veritas Corp., Tokyo, Japan) to produce the loading buffer. After aspirating the culture medium and leaving 2 mL, 3 mL of loading buffer was added and gently mixed. Cells were incubated at 37 °C with 5% CO_2_ for 15 min, followed by undergoing sequential aspiration and washing with medium to remove excess dye. Finally, 10 mL of maintenance medium was added to fully immerse the plate.

For fluorescence imaging, a white LED light source (X-Cite 110LED, Excelitas, Pittsburgh, PA, USA) with a wavelength range of 360–660 nm was filtered through a NIBA filter (470–490 nm, Olympus Corp., Tokyo, Japan) for excitation, with the emitted fluorescence (~518 nm) captured using a microscope camera (Retiga-2000R, QImaging, Surrey, BC, Canada), and both fluorescence movies and images recorded independently at 30 fps (movies) and 9 fps (images) with 200 × 150 pixels. Fluorescence intensity was used as a proxy for Ca^2+^ concentration. Nuclear staining of hiPSC-CMs was performed using a protocol similar to Fluo4 loading.

## 3. Results

**Figure 4:**
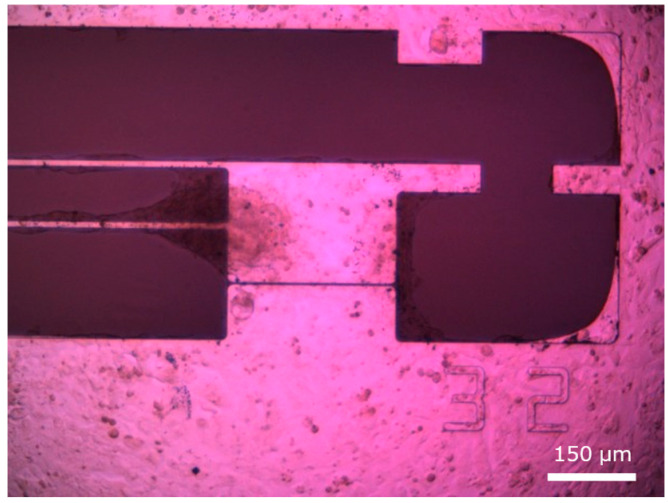
hiPSC-CMs seeded on the movable culture substrate.

The state of hiPSC-CMs seeded and cultured on the flexible substrate prior to fluorescent staining is shown in Figure 4. Cardiomyocytes adhered densely in both the movable and fixed regions, indicating successful cell culture, with microscopic observation revealing synchronous contractions of the cell clusters.

**Figure 5:**
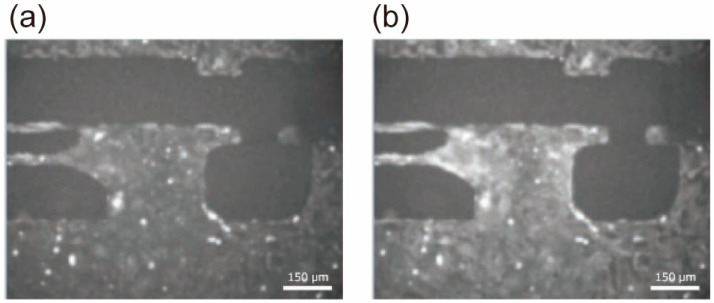
Fluorescent microscopic image of hiPSC-CMs during beating. The probe name is Fluo-4, with an excitation wavelength of 495 nm and an emission wavelength of 518 nm. The exposure time for imaging was set to 110 ms. (**a**) The moment when the hiPSC-CM cluster was relaxed; (**b**) the moment when the hiPSC-CM cluster was contracting.

Fluorescence microscopy images acquired after staining are presented in Figure 5. Fluorescence intensity varied significantly during the contraction cycle, reflecting changes in intra-cellular Ca^2+^ concentration, and fluorescence was dim when hiPSC-CMs were relaxed (Figure 5a) and increased during contraction (Figure 5b).

**Figure 6:**
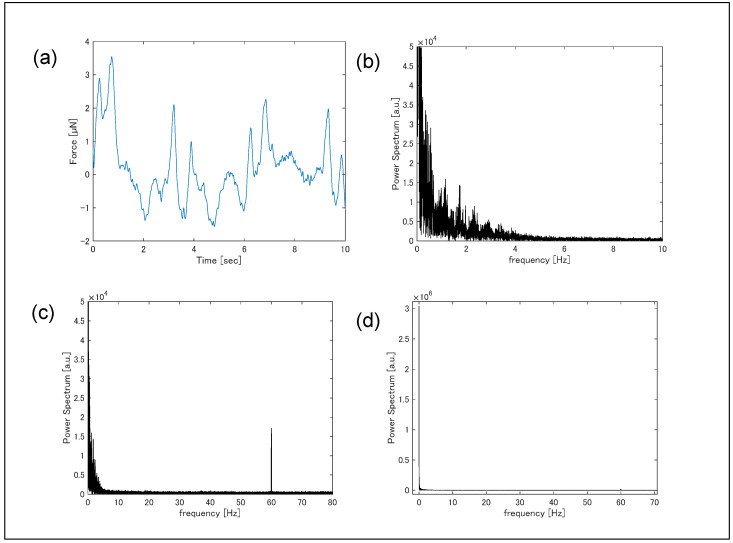
(**a**) Temporal variation in contraction force measured by the piezoresistive force sensor, and (**b**–**d**) fast Fourier transformation of the temporal variation.

Simultaneous measurements of contractile force were performed by monitoring substrate deformation during Ca^2+^ fluorescence imaging (Figure 6a). Force signals were sampled at 5 kHz, and cantilever deformation-induced resistance changes were converted to voltage using a Wheatstone bridge, amplified 1000-fold using a differential amplifier with a bandwidth of 1 Hz to 80 kHz, and recorded. While rhythmic contractions were observed, periods with no visible contractions (t = 1–3 s) indicated irregular timing, likely reflecting an arrhythmic state of the cardiomyocyte clusters potentially induced by fluorescent probe cytotoxicity. Fourier transformation of the time-dependent contraction data produced the frequency spectrum (Figure 6b). A signal was detected in the 0.4–3 Hz range, corresponding to contraction frequency. A sharp peak at 60 Hz was attributed to electrical line noise; in Toyama, Japan, the commercial power supply frequency is 60 Hz.

**Figure 7:**
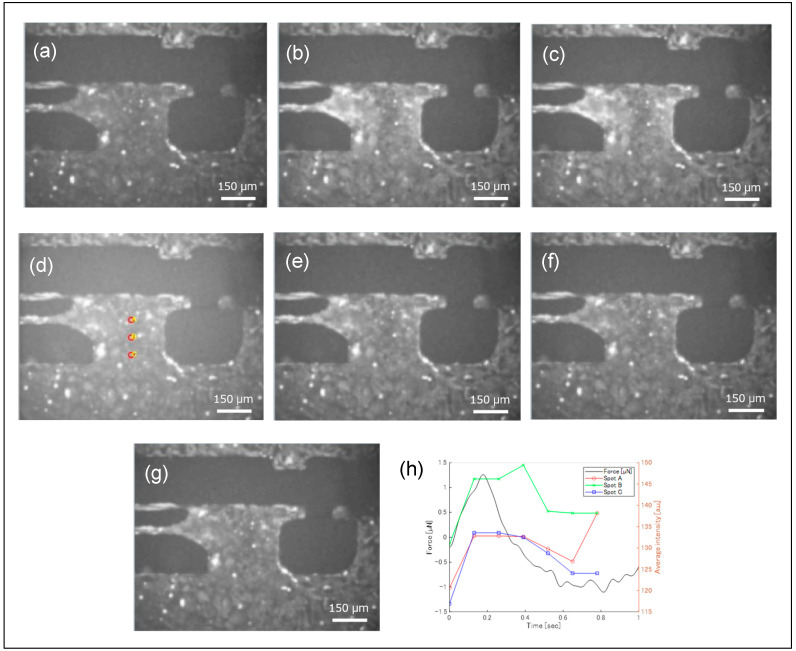
Fluorescent microscopic images of the hiPSC-CMs during a single cycle of beating, and temporal change in fluorescence intensity and a beating force. The fluorescence microscope images are arranged chronologically from (**a**–**g**). The fluorescence image intensities at points A, B, and C shown in (**d**) are displayed in the graph in (**h**).

Sequential Fluo-4 fluorescence images during contractions are shown in Figure 7, demonstrating that fluorescence intensity changed dynamically over time. Near the boundary between the movable substrate and arm, fluorescence variations were particularly pronounced. As indicated in Figure 4, this region contained a three-dimensional cluster of hiPSC-CMs, which likely did not contribute to substrate displacement. Nevertheless, synchronous blinking of this cluster with other cells was observed. While the pulsation force required only about 0.1 s for the signal to decay to approximately half its peak value, the fluorescence intensity took about 0.5 s to reach the same level.

The fluorescence intensities at three positions indicated in Figure 7d were extracted as pixel brightness and plotted over time (Figure 7), with corresponding contractile forces measured by a piezoresistive sensor shown in Figure 7h. Ca^2+^ levels and contractile forces increased nearly simultaneously; however, periods of reduced contractile force maintained relatively high Ca^2+^ levels. A measurement system capable of simultaneously performing pulsation force measurements and fluorescence observation can thus clearly demonstrate the temporal correlation between mechanical phenomena and chemical reactions.

**Figure 8:**
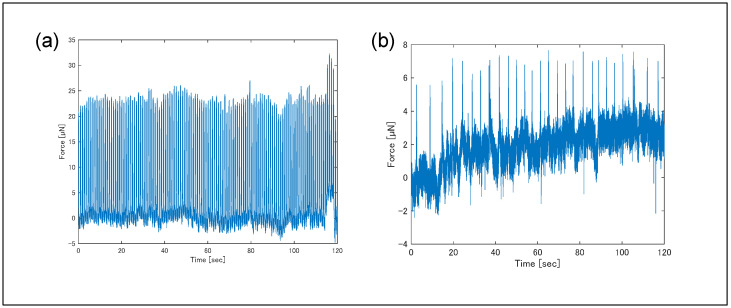
Temporal variation in contraction force measured (**a**) before and (**b**) after the fluorescent staining. Each measurement was performed multiple times; one example is shown here. Measurement temperatures were 36.4–36.5 °C for (**a**) and 36.0–36.8 °C for (**b**). Pulsatility was measured 14 days after seeding hiPSC-CMs onto plates. Fluo-4 (2–3 µM) was loaded for 15 min, 2 h after measuring (**a**), followed by measurement of (**b**). Pulsatility became unstable due to the influence of Fluo-4.

The effect of Fluo-4 staining on contractile behavior was evaluated by comparing forces before and after staining (Figure 8). Before staining, the average contractile force was 17 µN and the contraction frequency was 1.0 Hz; after staining, the average force decreased to 3.7 µN and frequency to 0.22 Hz, indicating a significant reduction. The decrease in contractile force after calcium staining may be attributed to the staining probe acting as an inhibitor, potentially hindering cross-bridge formation [31]. By binding to calcium ions, the probe likely reduced the amount of free calcium ions available for cross-bridge formation, resulting in fewer cross-bridges being formed. This reduction in cross-bridge formation is thought to have caused the decrease in contractile force.

Cases of reduced contractility following probe introduction have also been noted in the literature shown below. According to the article, byproducts of AM ester degradation, such as formaldehyde and acetic acid, are generated when the staining probe binds to calcium ions. These substances are reported to affect protein structure and induce a decrease in intra-cellular pH. This decrease in intra-cellular pH is noted to inhibit actin–myosin contractility and affect normal Ca^2+^ handling and maintenance of membrane potential.

**Figure 9:**
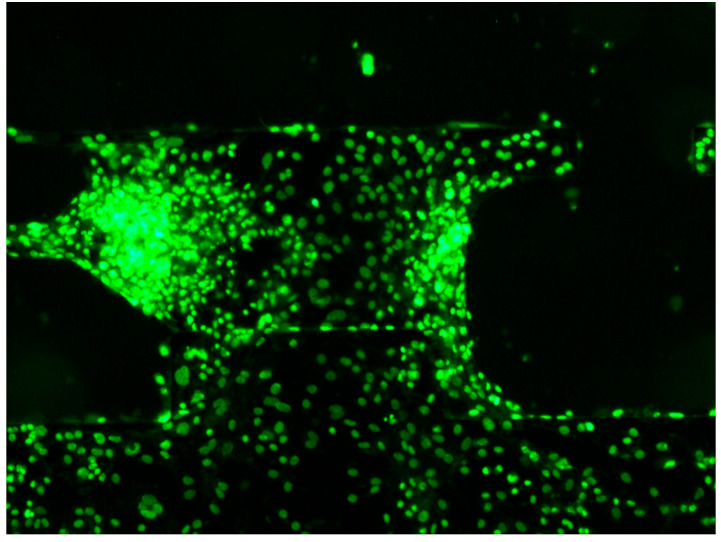
Images of hiPSC-CMs stained for their nuclei and observed under a fluorescence microscope. Multiple fluorescence imaging sessions were performed, and one example is shown here.

hiPSC-CM nuclei were stained and observed under fluorescence microscopy (Figure 9). Individual nuclei were clearly distinguishable, allowing cell positions and numbers to be determined. The white light source (X-cite) was set to 10% of maximum output, minimizing photobleaching and enabling continuous observation for up to 10 min. In this study, fluorescence imaging for each hiPSC-CM cluster was limited to approximately one minute, minimizing photobleaching effects. Some fluorescence signals in Figure 5 and Figure 7 were saturated, suggesting that a lower light intensity could enable longer-term imaging.

## 4. Discussion

In this study, we measured the contractile force and Ca^2+^ distribution of hiPSC-derived cardiomyocytes (hiPSC-CMs) simultaneously and in real time using a piezoresistive force sensor and fluorescence microscopy (Figure 1). For force measurement, the sensor was sampled at 5 kS/s, although the sensor system was capable of higher acquisition rates of up to approximately 100 kS/s. Serving as the force sensor, the cantilever was mounted on a piezo stage, enabling two-dimensional movement and allowing mechanical stretch to be applied to beating cardiomyocytes. Fluo-4 was used as a fluorescent probe for Ca^2+^ imaging, and temporal changes in fluorescence intensity across the entire field of view were observed in response to cardiomyocyte contractions. These intensity changes were considered to reflect intra-cellular Ca^2+^ dynamics. Additionally, nuclear staining of hiPSC-CMs seeded on a movable culture substrate allowed for visualization of individual cells.

By combining piezoresistive force measurement with Fluo-4 fluorescence imaging, the system was expected to directly quantify the temporal correlation between contractile force and Ca^2+^ concentration. In adult cardiomyocytes, the dynamics of Ca^2+^ cycling during contraction are increasingly well understood [23]. Membrane depolarization allows a small influx of Ca^2+^, which triggers a large release of Ca^2+^ from the sarcoplasmic reticulum. The elevated intra-cellular Ca^2+^ activates contractile proteins, inducing cell contraction. Subsequently, Ca^2+^ is re-sequestered into the sarcoplasmic reticulum or extruded via plasma membrane Ca^2+^ pumps, completing Ca^2+^ cycling. In contrast, hiPSC-CMs are relatively immature, exhibiting a low expression of Ca^2+^-handling genes, delayed Ca^2+^ signal decay, and reduced global Ca^2+^ synchrony. Overcoming this immaturity is an active area of research aimed at enabling hiPSC-CMs to be used in regenerative medicine [3,4,5,6].

Cardiomyocyte maturation has traditionally been assessed via structural observations (e.g., sarcomeres and gap junctions), electrophysiological measurements (e.g., action potentials and currents), and molecular biology approaches (e.g., gene expression). However, because the heart’s fundamental function is to act as a pump, it is desirable to directly evaluate the mechanical properties of cells. Techniques for precise measurement of hiPSC-CM contractile force have been proposed, and the next step is to examine the dynamics between the cellular environment and contractile function. In this study, we focused on Ca^2+^ ions, which are crucial for cardiomyocyte function, aiming to experimentally elucidate these dynamics.

Here, we proposed a novel sensing system and methodology that combined piezoresistive force measurement with fluorescence microscopy to investigate the temporal correlation between hiPSC-CM contraction and Ca^2+^ concentration. Using this system, dynamic changes in the latter were quantitatively captured during contractile force measurement. The remaining challenges for system improvement include accurately relating fluorescence intensity to Ca^2+^ concentration and minimizing the potential adverse effects of fluorescent probes on hiPSC-CMs.

We also demonstrated that nuclear staining of hiPSC-CMs on a movable culture substrate enables individual cell identification. Quantifying the number of cells contributing to the sensor-measured contractile force is highly significant.

To compare with conventional techniques for measuring beat force and Ca^2+^ concentration, the performance of each measurement method is summarized in Table 1 [32,33,34,35].

In summary, we propose a contractile measurement system that simultaneously quantifies hiPSC-CM contractile force, identifies the number of contributing cells, and visualizes Ca^2+^ distribution. Future improvements, including calculating the absolute Ca^2+^ concentration, minimizing fluorescent probe effects on cells, and achieving automated cell-counting functionality, will enable a detailed investigation of the dynamic relationship between cardiomyocyte contraction and Ca^2+^ signaling. This measurement system represents a powerful tool for studies aimed at enhancing hiPSC-CM maturation.

## Figures and Tables

**Table 1:** Comparison of conventional technologies for measuring beat force and Ca^2+^ concentration.

	Temporal Resolution (Frame Rate)		Image Resolution
	Typical	Highest	
Optical Mapping /High Speed Camera	500–1000 fps	4000 fps	0.1–1 mm
Confocal Laser Scanning Microscopy	10–50 fps	kHz order (theoretically)	H: 0.2–0.5 µm V: 0.5–1 µm
Two-photon Microscopy	10–100 fps	kHz order	H: 0.3–0.6 µm V: 1–2 µm
Piezoresistive Force Sensor (this work)	2000 S/sec	1 MS/sec	N/A

## Data Availability

The original contributions presented in this study are included in the article. Further inquiries can be directed to the corresponding author.
